# Assessing the Effectiveness of the Coprecipitation Method in Synthesizing Magnetic Nanocomposites Based on Iron Oxides Coated with Hydroxyapatite

**DOI:** 10.1021/acsomega.4c05427

**Published:** 2025-04-03

**Authors:** Maria L. Muñoz-Leon, Luis F. Zubieta-Otero, Diego F. Coral, Claudia F. Villaquiran-Raigoza, Mario E. Rodriguez-García

**Affiliations:** † Ciencia y Tecnología de Materiales Cerámicos (CYTEMAC), Facultad de Ciencias Naturales, Exactas y de la Educación, Departamento de Física, Universidad del Cauca, Calle 2A 3N-111, Popayán, Cauca 190002, Colombia; ‡ Posgrado en Ciencia e Ingeniería de Materiales, Centro de Física Aplicada y Tecnología Avanzada, Universidad Nacional Autónoma de México, Campus Juriquilla, Querétaro, Qro. 76230, México; § Departamento de Nanotecnología, Centro de Física Aplicada y Tecnología Avanzada, Universidad Nacional Autónoma de México, Campus Juriquilla, Querétaro, Qro. 76230, México

## Abstract

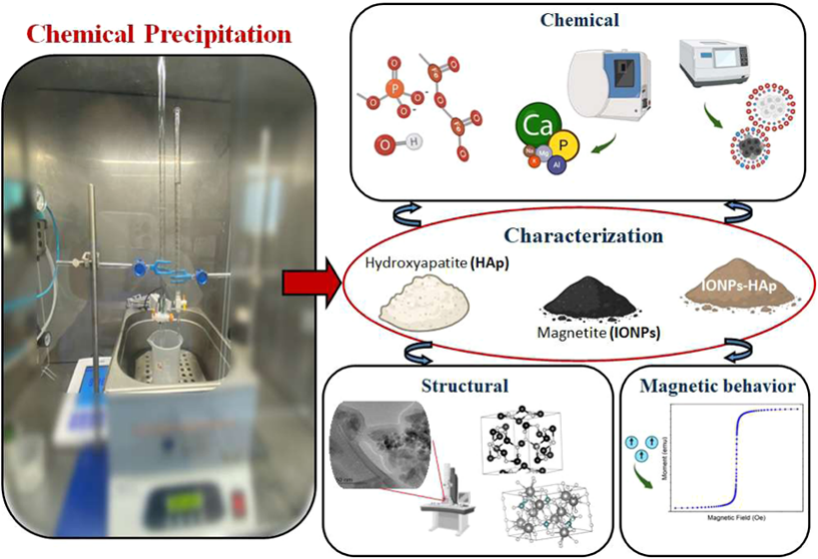

The aim of this work was the development and characterization of iron oxide nanoparticles with magnetite phases (IONPs)–hydroxyapatite (HAp) composites. In this article, the chemical coprecipitation method was used to synthesize three different nanomaterials: IONPs, HAp, and IONPs–HAp composite. Rietveld analysis of the X-ray diffraction (XRD) revealed the crystal lattice parameters and presence of HAp and IONPS after synthesis, which was carried out at a temperature of 120 °C inductively coupled plasma (ICP) was used to identify the trace elements present, Fourier transform infrared (FTIR) spectroscopy to verify the functional groups present in each material and efficiency of washes for the composite material, transmission electron microscopy (TEM) to observe the morphology and nanoparticle size for IONPs 11 nm and IONPs–HAp 15 nm. ζ potential measurements to investigate the surface charges for all samples had a positive value, the apatite samples showed a very stable behavior, and vibrating sample magnetometry (VSM) to evaluate the magnetic properties showed that IONPs and IONPs–HAp composite exhibit superparamagnetic behavior, while HAp nanoparticles show diamagnetic behavior. It was also shown that the saturation magnetization and magnetic moments of the IONPs do not change upon formation of the IONPs–HAp composite.

## 1 Introduction

Iron oxide nanoparticles with magnetite phases (IONPs) have been extensively studied due to their availability, versatility, and special magnetic properties. The most common iron oxides are maghemite, hematite, and magnetite. These oxides are usually formed as products of corrosion processes in ferrous structures, which are very common in nature. Magnetite is characterized by its excellent behavior in biological tests, its low toxicity compared to other iron oxides, and its unique magnetic properties in the size of nanoparticles, which makes it an extremely important material in the field of biomaterials. Particularly noteworthy is superparamagnetic behavior, which enables a fast and efficient response to external magnetic fields.^1^ This makes these nanoparticles suitable for biomedical applications such as magnetic hyperthermia,^1^ contrast agents,^2^ and drug delivery.^3^

However, there are still some challenges to overcome for biomedical applications such as their tendency to aggregate, cytotoxicity, and the determination of their chemical and colloidal properties or coating. Although IONPs can be easily produced by chemical methods, coatings need to be applied to prevent aggregation and improve their colloidal and chemical properties. For example, electrostatic, steric, or combined stabilization layers provide increased resistance to aggregation. A larger layer leads to higher chemical stability.^4^ However, it has been shown that the interaction between the coating and the surface of nanoparticles leads to magnetic disorder in the IONPs, reducing the effective magnetization of the nanoparticle. An inorganic matrix could be an effective coating material to create uniform, ultrafine, and dispersed nanostructures and produce a synergistic effect between the coating and the core.^5^ In this context, the potential applications of IONPs coated with SiO_2_ or gold were explored for photodynamic therapy and hyperthermia.^6^ In addition, IONPs have been coated with gadolinium to improve their potential use as contrast agents.^7^ This shows that a synergistic effect between the properties of IONPs and the coating can be achieved.

Hydroxyapatite (HAp) is the major inorganic component of the extracellular matrix based on calcium phosphate, which is abundant in mammalian bones and teeth.^8^ In its nanostructured form, HAp exhibits biocompatible and biodegradable properties and low water solubility and high stability under reducing and oxidizing conditions, and its synthesis is well-established.^9^ These properties have contributed to it being of great interest for biomedical applications such as the repair of hard tissue like bone.^10^ However, its mechanical properties are limited due to its inherent fragility. In this sense, HAp stands out as one of the most suitable inorganic materials due to its excellent results in biological tests. The combination of IONPs with HAp is advantageous because a functional surface enriched with phosphate groups has a high affinity for metal ions and achieves a strong electrostatic interaction,^11^ resulting in a stable and versatile composite.

When a HAp coating is applied to the IONPs, extremely beneficial results are achieved: First, the coating provides a potentially biocompatible surface that could reduce the toxic response in biological assays. Second, nanoparticles coated with this material can be gradually absorbed as hydroxyapatite is bioabsorbable, reducing the risks associated with excessive accumulation of IONPs.^12^ As a third advantage, HAp nanoparticles with magnetic properties induced by the presence of iron oxides are obtained. The magnetic character of the composite can be controlled from the synthesis stage using two different approaches: IONPs coated with HAp or by doping the crystal structure of HAp with Fe^2+^ and Fe^3+^ ions. The resulting physical properties depend on the type of approach used and are useful for biomedical applications.^7,13^

IONPs–HAp can be used as cell markers that magnetically transport osteoblasts into the injured region to enable tissue regeneration.^14^ This possibility has been demonstrated using stem cells loaded with IONPs to regenerate sciatic nerve injuries in rats.^15^ This type of material has also been shown to be useful in the controlled delivery of drugs, as they can improve the bioavailability of poorly soluble drugs due to their large surface area.^16^ In the case of hyperthermia, it has been demonstrated that it is possible to use this material to generate heat using radiofrequency magnetic fields, thus achieving a significant increase in the temperature of the medium in which the nanoparticles are located.^5^

Chemical coprecipitation has been widely used to synthesize IONPs and HAp nanoparticles.^17,18,20^ The coprecipitation of IONPs can be performed at room temperature or at temperatures below the boiling point of the solvent used for the reaction. The presence of an oxidative or inert atmosphere is required to obtain Fe_3_O_4_, γ-Fe_2_O_3_, or FeO. Due to their magnetic properties, it is necessary to obtain Fe_3_O_4_ or γ-Fe_2_O_3_ instead of FeO. In the case of HAp nanoparticles, as reported in ref (19), coprecipitation is carried out at temperatures below the boiling point of water, and in a second step, the sample is calcined at 650 °C to obtain crystalline HAp. Wet chemical coprecipitation was used to synthesize the IONPs–HAp composites. In this case, Ca^2+^ ions initiate nucleation on the surface of the IONPs due to their favorable ζ potential. Once coprecipitation is complete, the samples are dried but not calcined.^5^

Chemical coprecipitation is the preferred option for the synthesis of these materials as it can be produced easily and quickly, does not require high temperatures, is energy efficient, offers the possibility of surface modification of the particles, and achieves high homogeneity.^20^ In addition, coprecipitation is carried out at moderate temperatures and does not generate toxic products or byproducts.^21^ The absence of calcination results in HAp with low homogeneity and a tendency to agglomerate, which in turn inhibits the oxidative process from magnetite to hematite.^22^ The properties of HAp depend largely on the crystallinity, and the applications of IONPs depend on magnetic properties. Therefore, it is necessary to synthesize IONPs–HAp compounds with high crystallinity and a good magnetic response.

The aim of this work is to evaluate the effectiveness of the coprecipitation method in the synthesis of magnetic nanocomposites based on iron oxides coated with hydroxyapatite (HAp). The aim is to investigate and characterize how this method affects the homogeneity, crystallinity, and magnetic response of the nanocomposites, key aspects for biomedical applications such as magnetic hyperthermia, controlled drug release, and tissue regeneration.

## 2 Experimental Conditions

### 2.1 Synthesis of IONPs

For the synthesis of IONPs, iron chloride(II), FeCl_2_ (Sigma-Aldrich, 98%, Darmstadt, Germany), and iron nitrate(III), Fe(NO_3_)_3_·9H_2_O (Sigma-Aldrich, 98%, Darmstadt, Germany), were used as iron precursors in a molar ratio of 1:2 of Fe^2+^/Fe^3+^.^18^ First, the iron salts were dissolved in 100 mL of deionized water. Subsequently, the pH was adjusted by the dropwise addition of NH_4_OH (J.T. Baker, 28–30%, Madrid, Spain) to a pH of 10 at room temperature. The reaction is described as follows
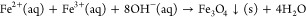
1The resulting suspension was centrifuged at 4000 rpm for 20 min and washed five times with deionized water to remove impurities and excess reactants. Finally, the obtained IONPs were dried at 120 °C for 5 h, removing water and residual ammonia, resulting in a fine brown powder.

### 2.2 Synthesis of Synthetic Hydroxyapatite

Hydroxyapatite was prepared using the wet chemical precipitation.^24^ A 0.6 M solution of (NH4)H_2_PO_4_ (99.1%, J.T. Baker, Mexico; code 0776-01) was added dropwise to a 1.0 M solution of Ca(NO_3_)_2_·4H_2_O (99%, Sigma-Aldrich, Japan; code 1002417639) while stirring and maintaining a temperature of 37.5 °C. The pH was adjusted to 9 using NH_4_OH (28.0–30.0%, J.T. Baker; code 9721-02) and an OAKTON pH meter (1100 series). After the reaction, the mixture was aged for 3 h, filtered to remove byproducts, and then heated at 250 °C for 3 h in a furnace. The synthesis was carried out maintaining a stoichiometric Ca/P molar ratio of 1.667. The process follows the reaction
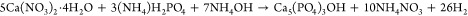
2

### 2.3 Synthesis of IONps–HAp

For the synthesis of the IONPs–HAp system, 1 g of the synthesized IONPs was taken and dispersed in a Ca(NO_3_)_2_·4H_2_O solution with a concentration of 1 mM under constant stirring. Then, a solution of NH_4_H_2_(PO_4_) at a concentration of 0.6 mM was added dropwise according to the same method as for hydroxyapatite synthesis. The resulting solution was aged for 1 h, after which NH_4_OH was added dropwise while maintaining a constant temperature of 40 °C.

This process was continued until a pH value of 9 was reached. The resulting solution was stirred for 2 h and aged for 48 h, washed, and filtered five times. Finally, the sample was dried in an oven at 120 °C for 2 h to remove byproducts.

### 2.4 Mineral Composition by Inductively Coupled Plasma Optical Emission Spectroscopy (ICP-OES)

The mineral content of the HAp and IONPs–HAp samples was analyzed using an inductively coupled plasma optical emission spectrometer (IGEO-UNAM, Queretaro, Mexico) (ICP-OES, Thermo iCAP 6500 Duo View). Each sample was digested with 0.1 g of nitric acid (Baker, 69–70% concentration). After digestion, the samples were returned to the ground state and heated in argon plasma. The elements were identified by their characteristic emission spectra, and their concentrations were determined by comparing the emission intensity to a reference curve.

### 2.5 X-ray Characterization

X-ray diffraction (XRD) technique was used to determine the crystalline phases. X-ray diffraction patterns were obtained using a Rigaku Ultima IV diffractometer (CFATA-UNAM, Queretaro, Mexico) operating at 40 kV, 30 mA, and a Cu Kα radiation wavelength of λ = 1.5406 Å. XRD data were collected in the 2θ range of 5–80° with a step size of 0.02°/min. LaB6 (NIST 660c) was used as the standard reference material (SRM) for calibration of the diffractometer line positions and line shapes. Diffraction patterns were analyzed by using GSAS-II software for Rietveld refinement.

### 2.6 Fourier Transform Infrared (FTIR) spectroscopy

IR spectroscopy was used to study the vibrational states of the functional groups. Spectra were performed using a PerkinElmer Spectrum Two (CFATA-UNAM, Queretaro, Mexico) equipped with an attenuated total reflectance (ATR) accessory with a diamond crystal in the spectral range from 600 to 4000 cm^–1^ at a spectral resolution of 2 cm^–1^.

### 2.7 ζ Potential Analysis

The ζ potential measurements (ζ, mV) were performed with the Anton Paar Litesizer 500 (CFATA-UNAM, Queretaro, Mexico) using omega cuvettes to measure the electrophoretic mobility of particles suspended in a liquid. IONPs, HAp, and IONPs–HAp samples were suspended in 96% ethyl alcohol. Measurements were repeated three times, at 25.0 ± 0.1 °C and pH: 7, using a 35 mW diode laser (λ = 658 nm) and a detection angle of 15°.

### 2.8 Transmission Electron Microscopy (TEM)

The microstructure of the samples was obtained using a JEOL ARM200-F transmission electron microscope (LUME-IIM-UNAM, Mexico City, Mexico) with an accelerating voltage of 200 kV. Samples were ultrasonically dispersed in isopropyl alcohol and extracted with a capillary tube to deposit a drop of the sample on a 3 mm copper grid and then placed in the TEM sample holder. Bright-field images and the array mask were applied to clean the fast Fourier transform (FFT) signal and calculate the interplane distances and were analyzed using Digital Micrograph software V4.0 from Gatan.

### 2.9 Magnetic Characterization

Magnetic analysis was conducted using a PPMS instrument in the vibrating sample magnetometry (VSM) (Universidad del Valle, Colombia) module at a temperature of 300 K and with a magnetic field sweep from −70 to 70 kOe. The specific magnetization (*M*) was found by normalizing the magnetic moment with the sample mass.

## 3 Results and Discussion

### 3.1 Mineral Composition

The trace elements detected in synthetic HAp included K (110 mg/kg), Na (126 mg/kg), Mg (224 mg/kg), and Al (945 mg/kg), while the main elements were Ca (350 046 mg/kg) and P (211 507 mg/kg). These trace elements may be incorporated into the HAp matrix either as substitutional atoms, such as Mg substituting for Ca, or as interstitial atoms. It is noteworthy that the inclusion of these trace elements in the HAp structure can alter the lattice parameters and result in shifts in the X-ray diffraction patterns of HAp. Additionally, the Ca/P ratio for this sample was 2.147, suggesting a deficiency of P and/or the substitution of P with other ions.

### 3.2 X-ray Diffraction Analysis

**Figure 1:**
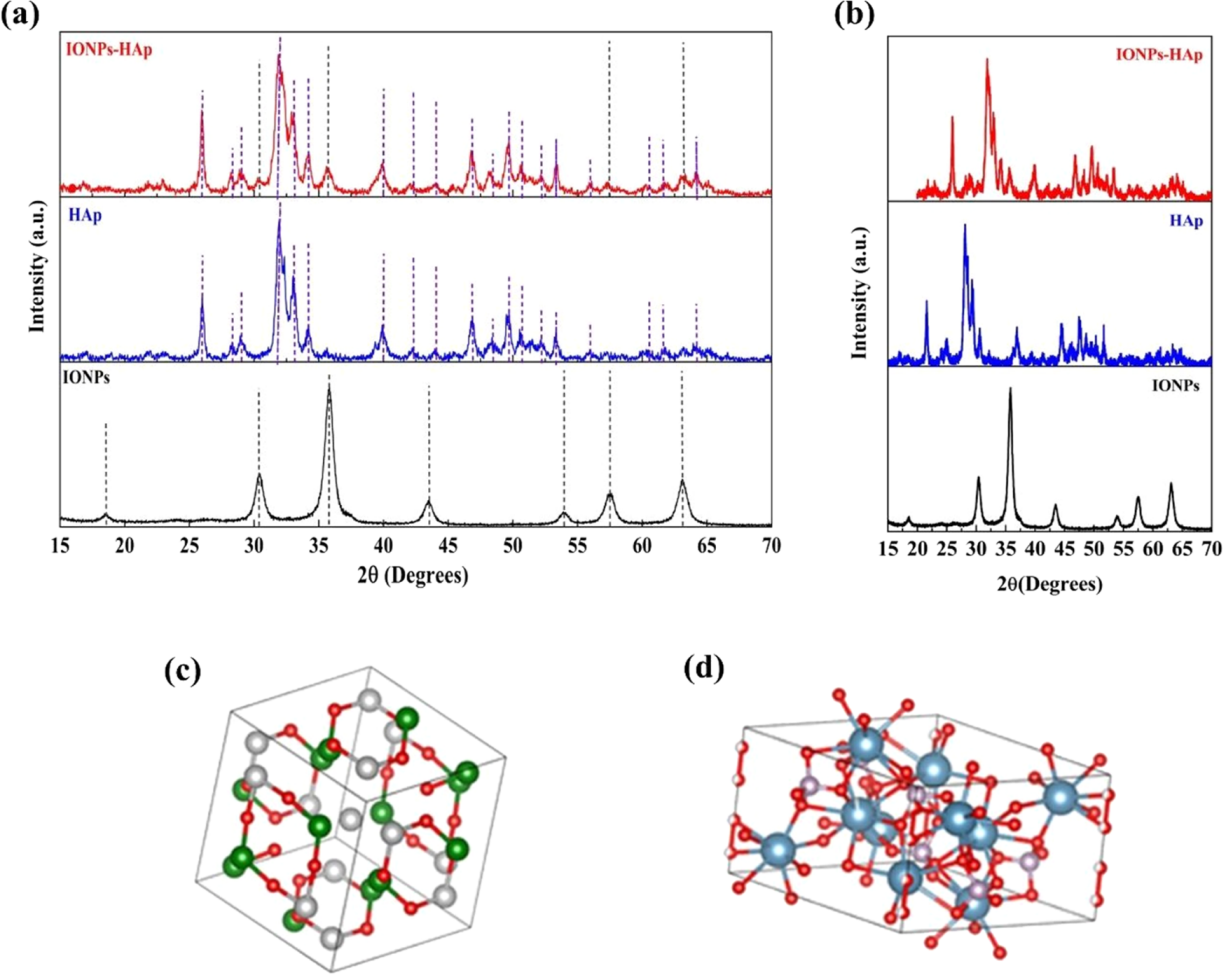
(a) X-ray diffraction patterns and (b) X-ray diffraction patterns Rietveld refinement obtained from the samples: IONPs, HAp, and IONPs–HAp. Crystal structures of (c) magnetite and (d) HAp constructed with the Rietveld refinement using Vesta software.

Figure 1 shows the diffraction patterns, Rietveld refinement, and crystal structures of the IONPs, HAp, and IONPs–HAp samples. Figure 1a shows the diffraction patterns of the IONPs, HAp, and IONPs–HAp samples. The diffraction peaks are consistent with PDF-ICDD card no. 00-019-0619 of magnetite (IONPs). For the HAp samples treated at 600 °C for 2 h, the diffraction peaks are consistent with PDF-ICDD card no. 00-009-0432^25^ of hydroxyapatite (HAp), and no secondary phases are observed. The diffraction peaks have a considerable width, indicating that the crystallite size is nanometric. Finally, it is also observed that the patterns of the IONPs–HAp composite, dried at 120 °C, contain magnetite and HAp in separate phases.

Table 1 shows the 2θ position and Miller indices for the diffraction peaks of magnetite, HAp, and IONPs–HAp. However, when analyzing the table, slight shifts to the right (>θ) of the peaks of the IONPs sample compared to HAp can be observed. These shifts could be due to the substituent ions of the synthesized HAp, as observed in the ICP results, where K, Na, Mg, and Al were found. Another hypothesis could be due to shrinkage of the unit cells due to residual stress or defects during coprecipitation.

**Table 1:** Identification of the Diffraction Peaks of IONPs, HAp and IONPs–HAp Samples Using the ICDD Card No. 00-019-0619 (Magnetite)^25^ and 00-009-0432 (HAp)^25^

IONPs		HAp				IONPs–HAp			
(*hkl*)	2θ	(*hkl*)	2θ	(*hkl*)	2θ	(*hkl*)	2θ	(*hkl*)	2θ
111	18.517	002	25.996	123	49.650	002^HAp^	25.958	222^HAp^	46.820
220	30.415	102	28.291	402	52.200	102^Hap^	28.200	312^HAp^	48.265
311	35.812	120	28.954	004	53.355	120^HAp^	28.924	123^HAp^	49.679
400	43.499	121	31.961	223	55.979	220^IONPs^	30.310	402^HAp^	52.200
422	53.946	112	32.343	331	60.400	121^HAp^	31.910	004^HAp^	53.355
511	57.494	300	33.032	214	61.775	300^HAp^	33.051	223^HAp^	55.979
440	63.122	202	34.208	304	64.035	112^HAp^	32.311	511^IONPs^	57.322
		310	39.909			202^HAp^	34.208	331^HAp^	60.440
		302	42.297			311^IONPs^	35.720	214^HAp^	61.700
		400	44.023			310^HAp^	39.929	440^IONPs^	63.175
		222	46.870			302^HAp^	42.285	304^HAp^	64.222
		312	48.421			400^HAp^	44.078		

Figure 1b shows the Rietveld refinement of the diffraction patterns from Figure 1a, which was performed with the GSAS-II software. The result obtained shows that the structure of IONPs is cubic with a lattice parameter of 8.3566 Å and an *Fd*3*m* space group, which represents a face-centered cubic structure by oxygen anions, typical of spinel and inverse spinel structures, with the presence of glide planes, mirror planes, and characteristic threefold rotation axes of the cubic structure. The sample density has a value of 5.287 g/cm^3^, and the χ^2^ value is 1.39. The Rietveld refinement of the diffraction pattern of HAp also shows that the structure is hexagonal and that the lattice parameters *a* and *c* have values of 9.4029 and 6.8723 Å, respectively. It has space group *P*6_3_/*m*, which is a primitive hexagonal structure, with the presence of helical axes with sixfold rotation axes and mirror planes, characteristic of the hexagonal structure. The sample density is 3.494 g/cm^3^, and the χ^2^ value determined was 1.77.

Rietveld refinement of the diffractogram of the IONPs–HAp compound showed the presence of two phases corresponding to magnetite and HAp. The IONPs have a cubic structure with a lattice parameter of 8.3467 Å and space group *Fd*3*m*; the percentage of this phase is 3.4% and its density is 5.289 g/cm^3^. HAp is the main phase with a percentage of 96.6% and has a hexagonal crystal structure with lattice parameters *a* and *c* of 9.4231 and 6.8762 Å, respectively, and space group *P*6_3_/*m*; its density was calculated to be 3.149 g/cm^3^ and χ^2^ = 1.85. Table 2 shows the lattice parameters of the IONPs, HAp, and IONPs–HAp samples obtained from the Rietveld refinement. It can be seen that the IONPs–HAp sample shows a slight increase in lattice parameters (*a* and *c*) compared to HAp. This indicates that the incorporation of IONPs exerts an influence that expands the crystal lattice in these directions, which also leads to a larger crystallite size.^26^

**Table 2:** Lattice Parameters for IONPs, HAp, and IONPs–HAp Samples Obtained from the Rietveld Refinement

	lattice parameters			
samples	*a* (Å)	*c* (Å)	density (g/cm^3^)	crystallite size (nm)
IONPs	8.3467		5.287	11.41 ± 0.08
HAp	9.4029	6.8723	3.494	10.02 ± 0.02
IONPs–HAp	9.4231	6.8762	3.149	18.53 ± 0.29

Figure 1c,d shows the construction of the magnetite and HAp structures using VESTA software from the results of the Rietveld refinement. Figure 1c shows the unit cell of magnetite, where the gray spheres represent Fe^2+^, the green spheres represent Fe^3+^, and the red spheres represent oxygen atoms. The crystal structure of HAp is depicted in Figure 1d, where the blue spheres represent calcium, the purple spheres represent phosphorus, and the red spheres represent oxygen atoms.

### 3.3 Vibrational Analysis

**Figure 2:**
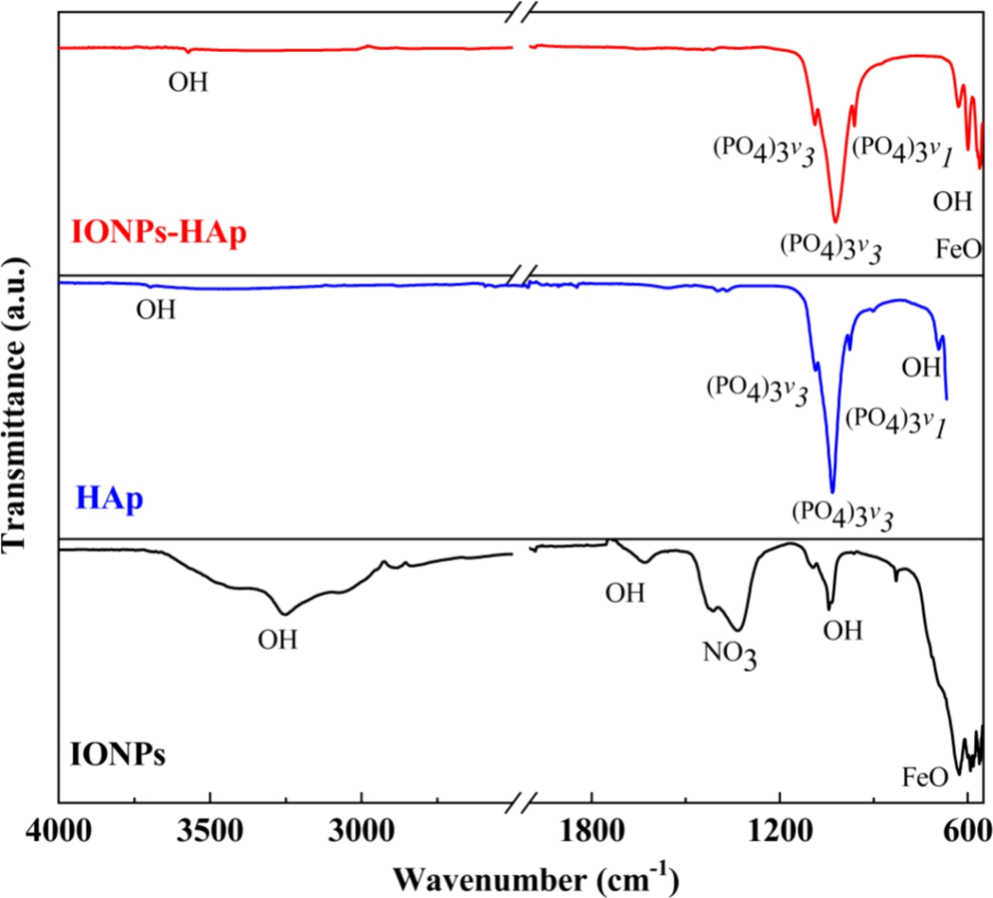
IR spectra of the IONPs, HAp, and IONPs–HAp samples.

Figure 2 and Table 3 show the infrared spectra and the position of the characteristic bands of the individual functional group of the IONPs, HAp, and IONPs–HAp samples. First, it can be seen that the IONPs sample has a characteristic band around 500–600 cm^–1^ associated with Fe–O binding.^26^ An absorption band of around 570 cm^–1^ was reported for bulk magnetite. The band at about 600 cm^–1^ is characteristic of the stretching mode of the Fe–O bond and may indicate the formation of Fe_3_O_4_. The band at approximately 589 cm^–1^ is characteristic of the Fe–O–Fe bond.^27^ In addition, the bands of OH and NO_3_ groups are observed in the IONP spectra, which correspond to the byproducts of the chemical reaction (eq 1).^28^ Due to the presence of byproducts in the IONPs sample, the sample was subjected to a drying process at temperatures above 120 °C. However, it was demonstrated that a phase transition occurs above 185 °C, due to the oxidation of magnetite, resulting in hematite.^29^ On the other hand, hydroxide functional group (OH) was detected around 1400 cm^–1^ and can be associated with water functional groups (H_2_O) adsorbed on the surface of the IONPs.^27^

**Table 3:** Observed Infrared Band Position for IONPs, HAp, and IONPs–HAp Samples^a^

IONPs–HAp			
functional group	wavenumber (cm^–1^)^b^	wavenumber (cm^–1^)^c^	references
O–H ν	3572	3571	(30) and (31)
PO_4_^3–^ ν_3_	1087	1090	(30) and (31)
	1022	1020	
PO_4_^3–^ ν_1_	961	960	(30) and (31)
O–H ν	629	625	(32)
FeO	584	570, 589	(26) and (27)

HAp			
functional group	wavenumber (cm^–1^)^b^	wavenumber (cm^–1^)^c^	references
O–H ν	3571	3571	(30) and (31)
PO_4_^3–^ ν_3_	1091	1090	(30) and (31)
	1026	1020	
PO_4_^3–^ ν_1_	960	960	(30) and (31)
O–H ν	630	625	(32)

IONPs			
functional group	wavenumber (cm^–1^)^b^	wavenumber (cm^–1^)^c^	references
O–H	3450	3448	(27) and (29)
O–H	1631	1635	(27) and (29)
	1030	1016	
NO_3_	1418	1426	(28)
	1334	1350	
FeO	586	570, 589	(26) and (27)

a ν = stretching, δ = scissoring, ρ = rocking, ω = wagging, τ = twisting.

b This work.

c References.

Figure 2 also shows the infrared spectrum of the HAp sample. The sample was heat treated at 250 °C for 2 h to remove all residues of the reagents used. The spectrum shows three different vibrational modes corresponding to the PO_4_^3–^ group. The strongest signal, corresponding to the stretching antisymmetric vibration (υ_3_), is at ∼1088 and ∼1022 cm^–1^. The symmetric stretching vibration mode (υ_1_) is at ∼960 cm^–1^, and around ∼600 and ∼560 cm^–1^, the phosphate group shows an asymmetric bending vibration mode (υ_4_).^30,31^ The characteristic hydroxyl group (OH−) of hydroxyapatite shows a bending vibrational mode around ∼630 cm^–1^. Considering the results in the infrared spectra of IONPs and HAp, which show the presence of functional groups corresponding to the precursors used in the synthesis process, it was decided to subject the samples of the IONPs–HAp system to a washing process with deionized water and dried at 120 °C for 2 h in an oven to remove byproducts. The infrared spectra of IONPs–HAp show that the byproducts of the synthesis processes (nitrate and carbonate groups) were eliminated. However, due to the concentration of HAp in the system, the characteristic bands of magnetite (IONPs) were masked.

### 3.4 Morphology by TEM

**Figure 3:**
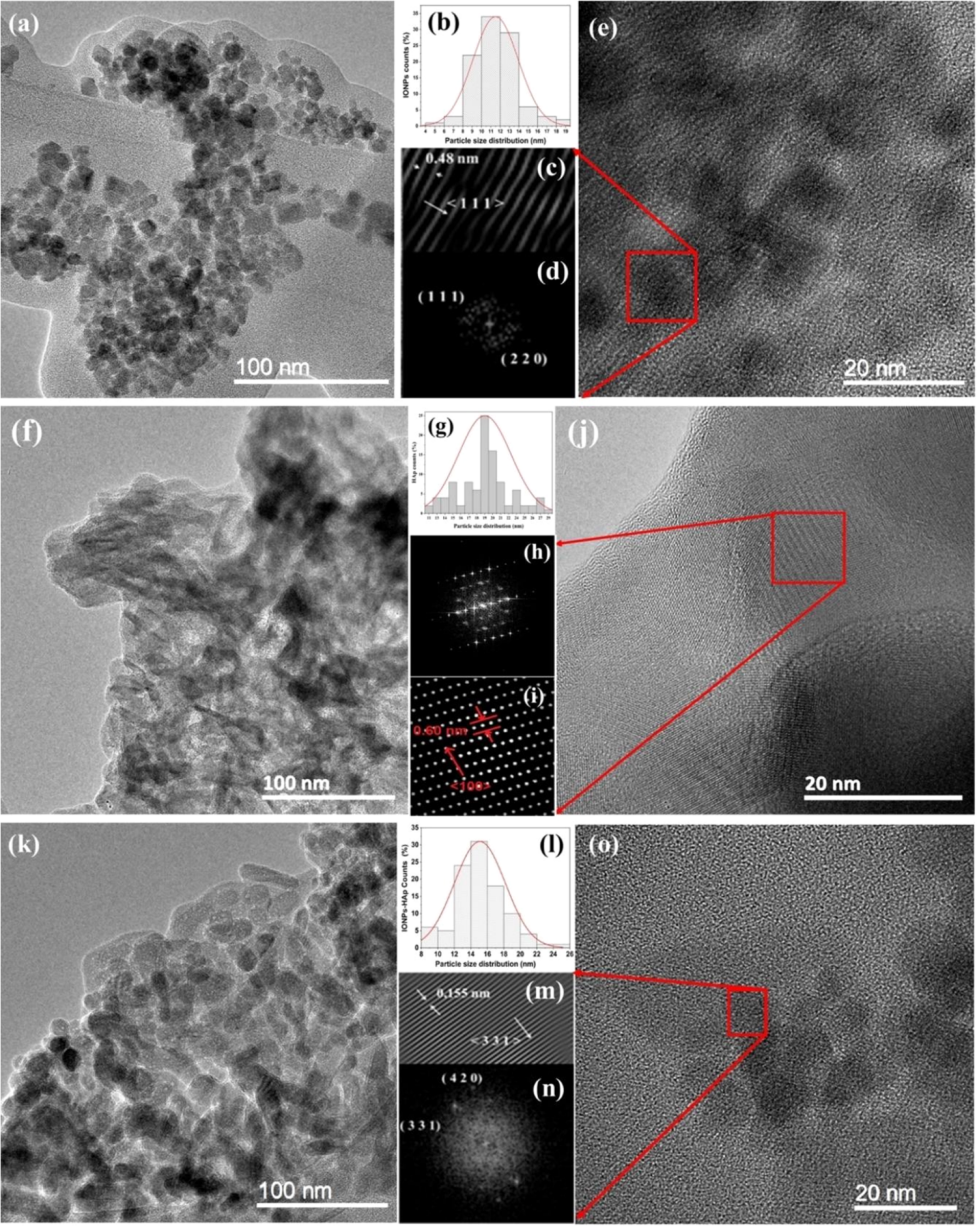
TEM micrographs, histogram particle size distribution, and fast Fourier transform (FFT) of the red section to determine the families of planes and interplanar distance of the samples: (a–e) IONPs, (f–j) HAp, and (k–o) IONPs–HAp.

Figure 3 shows the TEM micrographs, the histogram of particle size distribution obtained with ImageJ software, and the fast Fourier transform (FFT) of the red section to determine the plane families and interplanar distance obtained with Digital Micrograph V4.0 software of the IONPs, HAp, and IONPs–HAp samples. Figure 3a–e shows the cubic morphology of magnetite, with an average particle size distribution of 11 nm. Furthermore, the interplanar distance was found to be 0.480 nm, which corresponds to the magnetite plane (111) according to ICDD card No. 00-019-0619.^25^ The TEM results show a particle size distribution of about 11 nm, while the Rietveld refinement of the XRD data gives a crystallite size of 11.41 nm. This correlation could indicate that the synthesis process used favors the formation of individual particles, each consisting of a single crystallite. This could be due to the controlled growth conditions, such as homogeneous nucleation and uniform crystal growth.

Figure 3f–j shows the synthesized hydroxyapatite, which has a flake-like morphology with a variable particle size distribution due to the thickness of the wall. However, they are in the nanometer range between 10 and 30 nm. The distance found between the planes was 0.600 nm, indicating that we have a hexagonal structure that corresponds to the plane (100) according to ICDD card no. 00-009-0432.^25^Figure 3k–o shows the IONPs–HAp composites, where it can be observed that hydroxyapatite partially envelops some magnetite particles, and some degree of amorphicity is also observed due to the low temperature during heat treatment. Compared to the HAp sample, the hydroxyapatite in the composite (IONPs–HAp) is different because the magnetite can serve as nucleation sites to change its growth shape into polygons. The particle size distribution was 18 nm, and the interplanar distance found was 0.155 nm, which corresponds to the IONPs phase of the (331) plane family.

### 3.5 ζ Potential

**Figure 4:**
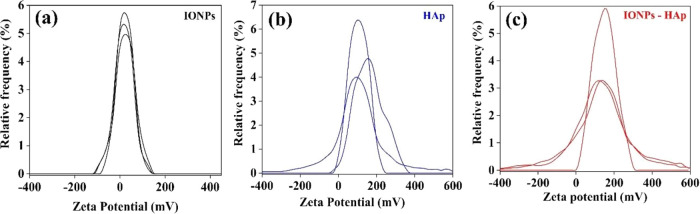
ζ potential of the samples: (a) IONPs, (b) HAp, and (c) IONPs–HAp.

To determine the surface charge of the samples obtained, their ζ potential was measured, performing three measurements for each sample. Figure 4a shows the measurements of the IONPs sample. A trend toward positive values can be seen from the figure, with a small portion showing a negative charge. It is expected that the IONPs have a negative surface charge.^33,34^ However, the positive potential of the synthesized samples can be attributed to the adsorption of ammonium ions during the synthesis process; these ions have a positive charge and could adhere to the magnetite nanoparticles. The average ζ potential of the IONPs was +21 mV, indicating that the particles tend to agglomerate.^35^ This assumption is supported by the FTIR results, where the presence of functional groups different from those characterizing the Fe–O bonds was observed.

The measurements of the ζ potential for the HAp sample are shown in Figure 4b, where predominantly positive behavior is observed. This result is like that obtained by other authors.^23^ The higher values of the ζ potential indicate the stability of the solution. The most important ions influencing the potential of HAp are H^+^, OH^–^, PO_4_^3–^, Ca^2+^, and the ions formed by their reactions. Negatively charged ions such as OH^–^, HPO^2–^, and HPO^4–^ therefore determine the net negative charge. The positive potential in the steady state, both in acidic and alkaline solutions, is probably due to the presence of Ca^2+^, CaOH^+^, and CaH_2_PO^4+^ on the surface.^36^

According to the ζ potential graphs of both IONPs and HAp, both systems have a positive ζ potential. Consequently, one would expect no coating to form, as both systems tend to repel each other. However, a rigorous washing process is sufficient to remove synthesis residues from the IONPs and obtain an IONP-HAp composite material. The ζ potential of IONPs–HAp is shown in Figure 4c, with positive values indicating that the solution is quite stable. Hydroxyapatite has sites with phosphate groups that carry a negative charge, which allows a high affinity for various metal ions and promotes interaction with the iron nanoparticles.^27^ The presence of OH^–^ ions at the corners of the HAp unit cell may contribute to the attraction of metal cations.^37^Table 4 summarizes the ζ potential data measured for the three systems studied.

**Table 4:** ζ Potential, Particle Size, and Magnetic Properties of the Three Systems Obtained^a^

samples	measurement	ζ-potential	mean ζ-potential (mV)	*d*_mag_ (nm)	μ (μ_B_)	*M*_s_ (emu/g_sample_)
IONPs	M1	19.9	+21 ± 3	7.5	10 715 ± 89	55.1 ± 0.5
	M2	18.1				
	M3	24.9				
HAp	M1	155.0	+118 ± 35			
	M2	96.0				
	M3	102.0				
IONPs–HAp	M1	119.0	+137 ± 18	7.6	11 211 ± 115	4.1 ± 0.04
	M2	155.0				
	M3	136.0				

a *d*_xrd_ stands for crystallite size determined by XRD, *d*_TEM_ for the particle size measured by TEM, *d*_mag_ for the particle size determined from magnetic measurements, μ for the mean magnetic moment, and *M*_s_ is the magnetic saturation of the samples.

### 3.6 Magnetic Analysis

**Figure 5:**
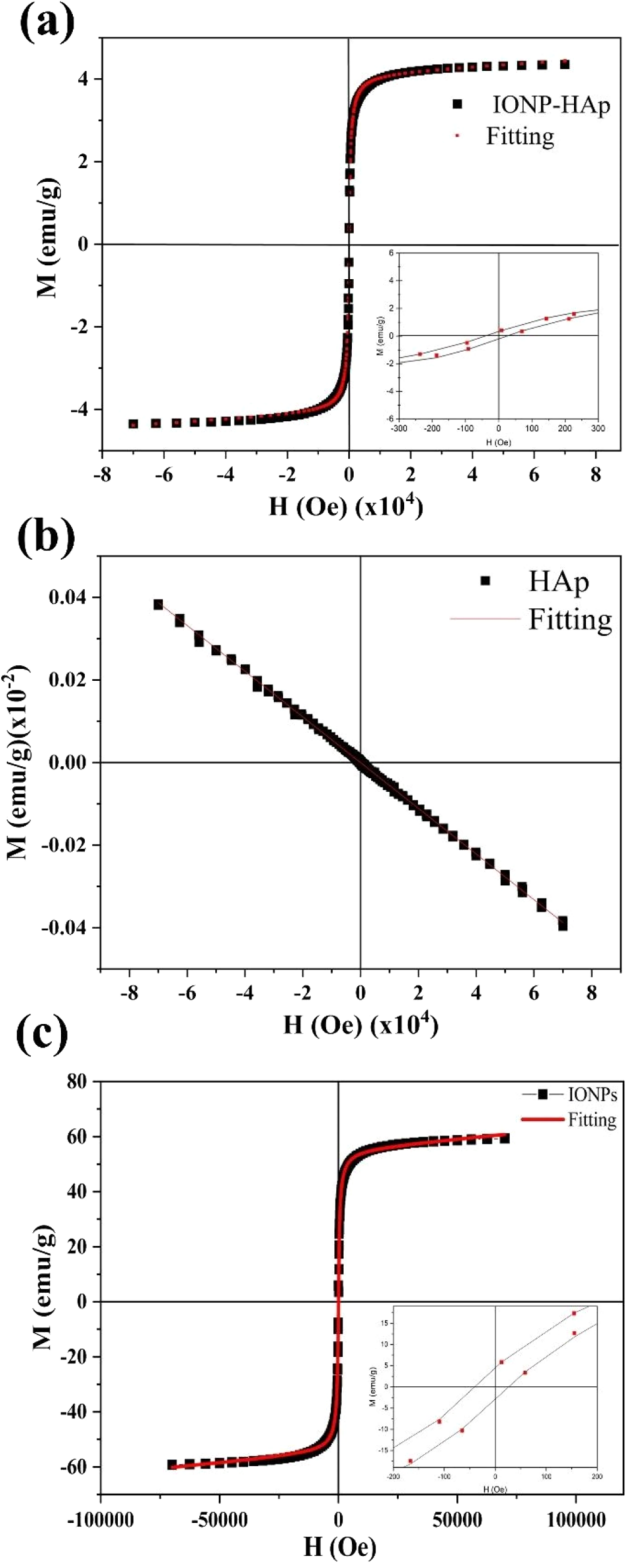
Magnetization as a function of external magnetic field for the systems: (a) HAp, (b) IONPs, and (c) IONPs–HAp; insets show an enlargement to highlight the material’s coercivity.

Figure 5 shows the magnetization (*M*) versus magnetic field (*H*) for the three samples obtained at room temperature. Figure 5a,c shows that the results of the IONPs and the IONPs–HAp sample are rapidly magnetized and reach saturation magnetization within the measured field range. In contrast, HAp (Figure 5b) shows a decrease in magnetization when the magnetic field is increased, which is a typical behavior for a diamagnetic system. The magnetic behavior of HAp is depicted in Figure 5b, showing linear behavior with a negative slope. The slope of the *M* vs *H* curve is related to magnetic susceptibility. The slope value of the linear fit is −5.533 × 10^–7^ emu/(Oe·g), consistent with expectations for a diamagnetic material; moreover, no saturation magnetization is observed in the case of HAp, as reported in the literature.^38^ To obtain the average magnetic moment value for samples IONPs and IONPs–HAp, the *M* vs *H* curves were fitted using a Langevin equation weighted with a log-normal distribution of magnetic moments *g*(μ) as shown in eq 1, where μ represents the magnetic moment of the particle, *N* is the number of particles in the sample, μ_0_ is the vacuum magnetic permeability, *k*_B_ is the Boltzmann constant, and *T* is the temperature.
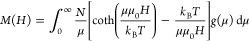
3The best fits are shown as continuous lines in Figure 5b,c. The correlation coefficient for both samples is 0.999, indicating that the samples exhibit a superparamagnetic behavior. Upon fitting, *M*_s_ = 55.1 ± 0.5 emu/g_sample_ was measured for the IONPs sample; this value is consistent with values reported in other studies. Notably, the value of saturation magnetization value exceeds the standard range of 10–30 emu/g commonly used in applications such as drug delivery^39^ and magnetic hyperthermia. On the other hand, the mean magnetic moment, reported as a multiple of the Bohr magneton (μ_B_), is μ = 10 715 ± 89 μ_B_, which is comparable to that reported in other studies for similar nanoparticles synthesized by the same method for magnetic hyperthermia applications.^26^ It has been reported that high crystallinity leads to a significantly higher magnetic moment^11,18^ which improves the properties related to biomedical applications.

In Figure 5, an enlargement of the region near the origin shows that the value of the coercive field of IONPs is Hc = 34.1 ± 0.5 Oe. For supermagnetic materials, zero coercivity is expected. A coercivity of zero is expected for supermagnetic materials, as observed for the IONPs, and could be explained by magnetic dipole interactions between the particles in the sample. This is important because it has been reported that higher coercivity leads to greater energy absorption by the magnetic system. This energy can be released as heat, resulting in a higher temperature increase, which is useful for magnetic hyperthermia treatment.^40^

Figure 5c shows the magnetic behavior of IONPs–HAp, which shows a significant decrease in magnetization compared to that of IONPs. The composite IONPs–HAp exhibits saturation magnetization values of 4.1 ± 0.04 emu/g_sample_, which corresponds to a reduction of 92.6% compared to IONPs. This reduction is related to the normalization of magnetization required to obtain specific magnetization. As the XRD analysis has shown, the IONPs–HAp sample has a low percentage of magnetic material; therefore, a mass that includes both the superparamagnetic and diamagnetic material is used in the normalization of the sample mass. On the other hand, the mean magnetic moment has a value of 11 211 ± 115 μ_B_ and a coercivity of 35.6 ± 0.04 Oe. These values are similar to those obtained for the IONPs sample, which allows two main conclusions to be drawn. The interaction with HAp does not alter the coercivity of the sample, suggesting that magnetic dipolar interactions are still present, possibly due to the coating of aggregates rather than individual particles.

However, the average magnetic moment of the particle is not affected, and there is no change in the effective saturation magnetization, which shows that the HAp coating does not affect the crystallinity of the magnetite nanoparticle and thus does not create a magnetic dead layer that reduces the magnetization of the particle as can be seen in Table 1 where the magnetic size calculations are presented, showing that the magnetic diameter (*d*_mag_) remains almost constant for both samples. Finally, it is also important to emphasize that despite the low concentration of IONPs (3.4%) in the IONPs–HAp sample, this amount of magnetic material is sufficient to counteract the diamagnetic effect observed for HAp.

## 4 Conclusions

Crystalline nanoparticles of IONPs, HAp, and IONPs–HAp were obtained by the chemical coprecipitation method, all of which are free of secondary phases and have a nanoscale size. From the ζ potential values obtained, HAp imparts stability to the solution containing IONPs and HAp and facilitates the adhesion of HAp to the surface of IONPs, so this composite was obtained at 120 °C with nanometer size and a certain degree of amorphicity. The superparamagnetic behavior and saturation magnetization make the IONP-HAp system a promising biomaterial for biomedical applications. This research represents a significant advance in the field of synthesis, characterization, and invention of new materials with the desired magnetic, structural, and morphological properties. By use of a chemical method that does not require aggressive conditions, innovative materials with potential applications in various technological fields have been developed. The results obtained emphasize the effectiveness and versatility of the proposed method and open new possibilities for the development of nanocomposites and other advanced materials in the near future.

## Data Availability

The data underlying this study are not publicly available due to they are unpublished data for another study.
